# Antifungal Susceptibility Testing of *Fusarium*: A Practical Approach

**DOI:** 10.3390/jof3020019

**Published:** 2017-04-26

**Authors:** Abdullah M. S. Al-Hatmi, Ilse Curfs-Breuker, G. Sybren de Hoog, Jacques F. Meis, Paul E. Verweij

**Affiliations:** 1 Westerdijk Fungal Biodiversity Institute, PO Box 85167, 3508 AD Utrecht, The Netherlands; s.hoog@westerdijkinstitute.nl; 2 Centre of Expertise in Mycology Radboud University Medical Centre, Canisius Wilhelmina Hospital, 6500HB Nijmegen, The Netherlands; jacques.meis@gmail.com (J.F.M.); Paul.Verweij@radboudumc.nl (P.E.V.); 3 Ministry of Health, Directorate General of Health Services, PO Box 393, 100 Muscat, Oman; 4 Department of Medical Microbiology and Infectious Diseases, Canisius Wilhelmina Hospital, 6500HB Nijmegen, The Netherlands; i.breuker@cwz.nl; 5 Basic Pathology Department, Federal University of Paraná State, Curitiba, 81540-970 Paraná, Brazil; 6 Biological Sciences Department, Faculty of Science, King Abdulaziz University, PO Box 80203 Jeddah, Saudi Arabia; 7 Department of Medical Microbiology, Radboud University, Nijmegen Medical Centre, 6500GS Nijmegen, The Netherlands

**Keywords:** *Fusarium*, susceptibility test, clinical laboratory, CLSI, EUCAST, Etest

## Abstract

In vitro susceptibility testing of *Fusarium* is becoming increasingly important because of frequency and diversity of infections and because resistance profiles are species-specific. Reference methods for antifungal susceptibility testing (AFST) are those of Clinical and Laboratory Standards Institute (CLSI) and European Committee on Antimicrobial Susceptibility (EUCAST), but breakpoints (BPs) have not yet been established. One of the problems is that phylogenetic distances between *Fusarium* species are much smaller than between species of, e.g., *Candida*. Epidemiological cutoff values (ECVs) for some *Fusarium* species have been determined in order to differentiate wild-type from non-wild-type isolates. In clinical routine, commercially available assays such as Etest, Sensititre or others provide essential agreement with reference methods. Our objective is to summarize antifungal susceptibility testing of *Fusarium* genus in the clinical laboratory: how to do it, when to do it, and how to interpret it.

## 1. Introduction

Species of the genus *Fusarium* are involved in a gamut of human infections. Keratitis and onychomycosis are prevalent in immunocompetent individuals [1]. Mycotic keratitis is a worldwide important ophthalmic problem causing visual disability due to its protracted course and unfavorable response. In half of the cases in endemic areas like the South of India, the filamentous fungi *Aspergillus* and *Fusarium* are the most common species causing keratitis [2] while immunocompromised hosts with hematological malignancies and those subjected to solid organ transplant, allogeneic bone marrow transplant, or peritoneal dialysis [3,4] are at risk of disseminated, frequently fatal infections. Fungemia in severely compromised patients leads to local necrosis after secondary cutaneous dissemination. In very rare cases, *Fusarium* can also cause skin infections characterized by small, painless papules which quickly progress to painless ulcers with black eschars [5].

Delayed initiation of antifungal therapy due to non-optimal diagnosis of mold infections are associated with increased mortality rates and may lead to excessive drug use in prophylaxis and therapy [1,6]. The *Fusarium solani* species complex contains many opportunistic species (e.g., *F. falciforme*, *F. keratoplasticum* and *F. petroliphilum*) with high prevalence, but other *Fusarium* groups are also important, such as *F. oxysporum*, *F. verticillioides* and *F. proliferatum*, and susceptibility to antifungal agents varies between species [7]. Treatment strategies for fusariosis have been evaluated [8] which led to the European Society of Clinical Microbiology and Infectious Diseases (ESCMID) ESCMID and European Confederation of Medical Mycology (ECMM) guidelines for management of hyalohyphomycosis caused by *Fusarium* and other non-melanized fungi [9]. Multiresistance to antifungals, observed in all *Fusarium* species, is intrinsic therefore these fungi are notoriously difficult to treat. 

*Fusarium* species do not have a normal minimum inhibitory concentration (MIC) and minimum effective concentration (MEC) distribution [10,11] and therefore prediction of antifungal susceptibility of a single strain is difficult. However, at least the species show different tendencies in their susceptibility against various antifungal compounds [11]. Susceptibility testing should be included in the routine patient management to optimize appropriate therapy, particularly in severe infections. From an epidemiological point of view, little is known about the prevalence of resistance in *Fusarium* infections, due to the fact that most of the laboratories do not routinely perform antifungal susceptibility testing and also because many laboratories face difficulties in correctly identifying species of *Fusarium*. 

The role of the clinical microbiology laboratory is important to confirm susceptibility to the chosen empirical antifungal agents, or to detect resistance in individual *Fusarium* isolates. From a practical perspective, clinicians often perceive such test results to be at least as important as the identity of the etiologic agents. The goal of this article is to provide a review of current concepts in laboratory methods and approaches of antifungal susceptibility testing that serve to assist clinicians in making optimal antifungal decisions for treatment of fusariosis.

## 2. *Fusarium* Species Identification in the Clinical Setting

Accurate identification of *Fusarium* species from human samples down to species level is important not only from an epidemiological viewpoint, but also for choosing the appropriate antifungal treatment. However, rapid identification of *Fusarium* isolates from clinical samples is particularly important given their innately variable antifungal susceptibility profiles, mainly for amphotericin B, voriconazole and posaconazole [4,11], but is complicated by the absence of diagnostic morphological features and also increasing number of emerging pathogenic species.

The genus *Fusarium* was divided into sections [12] but the current classification scheme replaces the designation “section” with “complex” [13]. Currently, the genus *Fusarium* is classified into 20 complexes that are comprised of related species [13]. Identification of *Fusarium* to the genus level is possible in the clinical microbiology laboratories relying mainly on morphology-based identification by recognizing macroscopic (colony appearance, texture/structure, pigmentation and colour of exudates) and microscopic (conidiogenous cells, (type and size of conidia) and type of conidiogeneisis). However, difficulties exist when using phenotype-based scheme because these characteristics are unstable and clinical *Fusarium* sometimes manifest atypically with sporulation. Furthermore, members of the same complex have overlapping morphological characteristics, with several genetically distinct species existing within the genus *Fusarium*.

Clinically, identification of unknown *Fusarium* clinical isolates to species may be important given that different species have variable susceptibilities to multiple antifungal drugs. Thus, knowledge of the species identity may influence the choice of appropriate antifungal therapy. The use of molecular genetic data appears to be essential to recognize monophyletic *Fusarium* species. Multilocus data have been applied to separate closely related taxa and provide support for species borderlines in *Fusarium* [14] as successfully used in other fungi. DNA sequencing of partial genes have been used to supplement morphological identification of *Fusarium* species. Use of the nuclear ribosomal internal transcribed spacer (ITS) sequence should be sufficient to place most isolates within the appropriate complex or *Fusarium* genus, but will not provide sufficient sensitivity to discriminate among individual species within the complex. Protein-coding genes are also in use, such as RNA polymerase (*RPB1/2*), β-tubulin (*BT2*), elongation factor (*TEF1*) [15]. *TEF1* has been widely used for species identification in *Fusarium*. Sequences for this gene are available through GenBank and through the more focused *Fusarium*-ID [16], or *Fusarium* MLST [17]. Some single-copy protein-coding genes such as *RPB1* and *RPB2* are also promising for *Fusarium* identification [13]. 

Another promising approach for the quick identification of *Fusarium* is matrix-assisted laser desorption ionization-time-of-flight (MALDI-TOF) [18]. MALDI-TOF MS uses species-specific patterns of peptides and protein masses to identify microorganisms. These peptides are converted into ions by either addition or loss of one or multiple protons [19]. Several studies have been conducted on *Fusarium* species with high success rates of 82%‒99% [20,21,22]. This method was performed in the *F. fujikuroi* species complex. A database was built as a result of this research which can be used as a future reference tool [18].

## 3. Technical Reference of Antifungal Susceptibility Testing Methods for *Fusarium*

Antifungal susceptibility testing is a very dynamic field of medical mycology. There are two recognized standard methods for the performance of antifungal susceptibility testing of *Fusarium* species that apply broth microdilution (BMD), i.e., the Clinical and Laboratory Standards Institute (CLSI) [23] and the European Committee on Antimicrobial Susceptibility Testing (EUCAST) [24]. These methods provide MIC data for all classes of antifungal agents that are both quantitatively and qualitatively comparable [25,26]. Alastruey-Izquierdo et al. [27] indicated that in vitro antifungal susceptibility testing plays an increasingly important role in guiding clinicians in giving the proper antifungals, in drug development programs, and for tracing the development of antifungal resistance in epidemiologic studies. Recently, Espinel-Ingroff et al. [28] established epidemiological cutoff values (ECVs) for some species in order to distinguish wild-type (wt; is the population of strains in a species/drug combination with no detectable acquired resistance mechanism) from non-wild-type (non-wt; agents with decreased susceptibility to a certain drug). Clinical interpretative breakpoints (CBPs) for in vitro antimicrobial susceptible testing for *Fusarium* are not yet established but epidemiological cutoff values (ECVs) for three *Fusarium* species have been suggested [28]. 

## 4. In Vitro Antifungal Susceptibility Testing

Several antifungal agents have been developed and are becoming available for clinical use [29]. Even though standardized in vitro susceptibility testing has been developed by CLSI and EUCAST yielding reproducible results, correlation with clinical outcome is still an enigma. The methods measure antifungal activity, expressed as the minimum inhibitory concentration (MIC) of an antifungal, which indicates the minimal concentration of drug able to inhibit fungal growth. Furthermore, both methods are useful to screen for resistant strains and determine the potential therapeutic value of a new antifungal agent [30]. Of note, several studies have shown that the in vitro results may be influenced by factors such as inoculum size, composition and pH of the medium, and incubation temperature and duration [31,32,33]. 

Establishing correlation between in vitro susceptibility tests and clinical outcome has been difficult [34]. According to the “90–60” Rule [35], infections due to susceptible isolates respond to therapy ~90% of the time, whereas infections due to resistant isolates respond to therapy ~60% of the time. Hence, antifungal susceptibility testing can predict the outcome of treatment only in main traits. Low MICs do not guarantee clinical success, while high MICs are associated with lower probability of a favorable response to a given antifungal agent. Applying this rule in *Fusarium*, the 90–60 rule reflects the fact that in vitro susceptibility of a given strain is only one of several factors influencing success of therapy. Despite the intrinsic resistance in *Fusarium*, in vitro testing remains useful in guiding clinicians in taking the right therapeutic decision because many *Fusarium* species show species-specific antifungal profiles [4,11].

## 5. Correlation between In Vitro and In Vivo

The MIC concept remains the only important parameter for defining antifungal activity and predicting antifungal potency against the fungus at hand. Despite the advantages that MICs offer in the clinical setting, a drawback is the lack of information that they provide concerning antimicrobial activity over time. It seems that prediction of the therapeutic success based on the MIC of a fungal strain is not always possible, because studies conducted until now have shown a poor correlation between the in vitro data with the in vivo outcome. For example, high MICs of itraconazole for a *Fusarium* strain isolated from a mycetoma case have been found, but the patient improved and completely recovered using itraconazole [36]. Various host factors affect the response to antifungal drugs, such as underlying disease, host immune function, catheter removal, surgical interventions, and pharmacokinetic parameters [37]. Molecular mechanisms of antifungal resistance have not been studied in *Fusarium*, however, molecular characterization of resistance mechanism and improvements of antifungal susceptibility testing methods is important [38].

## 6. EUCAST vs. CLSI

In vitro antifungal susceptibility profiles of *Fusarium* species demonstrate high MICs to most antifungal agents [10,11,39,40,41,42,43,44,45,46,47,48,49,50,51,52,53,54]. Notably, some species may exhibit different patterns of susceptibility: *F. solani* species complex are usually resistant to azoles and exhibit higher amphotericin B MIC values than other species, whereas *F. oxysporum* and *F. verticilloides* may be susceptible to voriconazole and posaconazole [10,11,55]. The echinocandins are not active against *Fusarium* spp. [4,11,54].

The CLSI and EUCAST are different from each other but in terms of MIC values, they are very similar when tested for azoles (posaconazole and voriconazole), and reading is performed after 48 or 72 h of incubation. The two methods differ in the amount of glucose present in the media (0.2% and 2%, respectively), fungal inoculum (10 times higher in EUCAST), in the type of microtiter wells used (flat bottom versus “U” shaped) and in the concentration of DMSO (0.5% and 1%, respectively). Reading of the microliter plate is done by spectrophotometric for EUCAST and visually for CLSI. Despite some differences in inoculum size, medium composition and well shape, the two standards have proved to yield comparable results. Both methods introduce the concept of Minimum Effective Concentration (MEC) for reading echinocandin results: transition point of hyphae from normal to aberrant hyphae, evidenced by appearance of granules or clusters on the bottom of the wells of the microplates, although this is not always easy to see and may require microscopic observation. Reading of the MEC, EUCAST indicates that there are still no reliable methods for the study of the sensitivity of fungi to echinocandins [24]. Assuring quality of test performance and results (reagents, inoculum and procedure) recommended reference strains are included. EUCAST recommended, among others, the strains *A. fumigatus* ATCC 46645, *A. flavus* ATCC 204305 and ATCC 204304. CLSI recommended, among others, the use of *Paecilomyces variotii* ATCC MYA 3630 and *A. flavus* ATCC 204304. 

Good correlation is obtained between MICs from CLSI and EUCAST for amphotericin B, voriconazole and posaconazole among *Fusarium* species [56], with categorical agreement (CA) of 100%, 95% and 75%, respectively, differing within ±2 dilutions [56]. In general, the MICs for amphotericin B spanned a range of 0.25 to 8 μg/mL with the CLSI and the EUCAST methods and in a wider range of 0.25–32 for voriconazole and posaconazole with both methods.

## 7. Etest and Other Commercial Methods

The Etest method is based upon the establishment of a stable concentration gradient of an antimicrobial agent following diffusion from a plastic strip into an agar medium. When an Etest strip is placed upon an agar plate that has been inoculated with a test organism and incubated for 24 to 48 h, an ellipse of growth inhibition occurs, and the intersection of the ellipse with the numeric scale on the strip provides an indication of the MIC. MICs determined by Etest generally agree with those determined by CLSI and EUCAST methods; however, this agreement may vary depending upon the antifungal agent tested, the choice of agar medium, and the fungal species [56,57]. Al-Hatmi et al. [56] reported that the overall agreement between the CLSI, EUCAST and Etest for drugs tested (amphotericin B, voriconazole and posaconazole) was slightly higher with EUCAST (90–100%) than with CLSI (85–95%). Etest thus can be considered as an appropriate method to determine the resistance in vitro to amphotericin B, voriconazole and posaconazole. For echinocandins, insufficient comparative data are available. Other commercially available alternatives have been developed, often employing a microdilution format and spectrophotometric or colorimetric reading [58]. These methods include Sensititre YeastOne^®^ (bioMerieux S.A., Marcy-l’Étoile, France) as well as panels for disk diffusion. Most of these tests have not been validated for antifungal susceptibility testing of *Fusarium*.

## 8. When Should AFST Be Done for *Fusarium*?

According to the different clinical experiences, susceptibility testing of *Fusarium* should be done to optimize the therapy of the individual patient or for epidemiological purposes. It is recommended to perform the MIC if the fungus has been isolated from sterile biological sites, in case of deep infection in patients undergoing antifungal therapy, in the event of therapeutic failure, when the isolated species is rare and/or emerging or in case of a particular species for which there is the suspicion that it may be resistant or less sensitive to the employed antifungal [9]. 

## 9. Protocol

**Figure 1:**
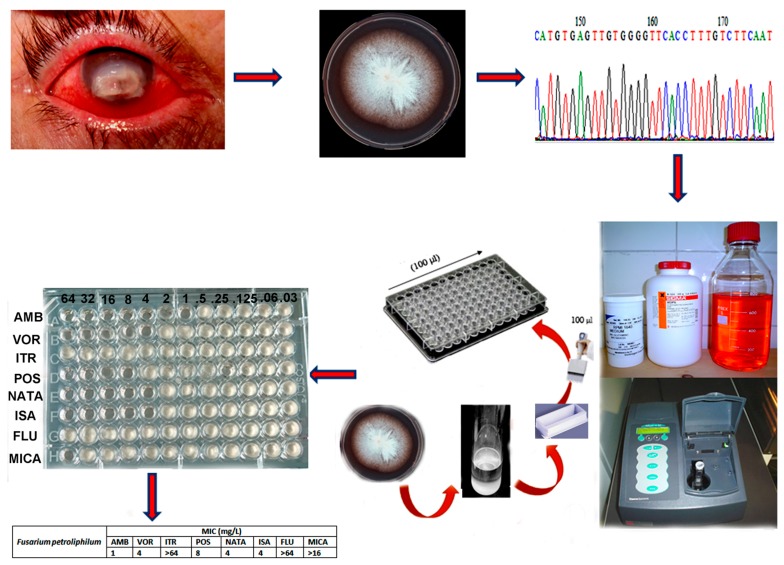
Antifungal susceptibility testing workflow.

Each laboratory needs to perform a risk assessment in collaboration with their Institutional Biological Safety Officer to determine the appropriate biosafety level for preparation of the inoculum and for pipetting of the inoculum into the plate. For *Fusarium*, biosafety level-2 laboratory (BSL-2) practice is required. Inoculum and microdilution plates are placed inside a plastic bag during the incubation at 35 °C. There are relatively few drugs to treat fusariosis. Below the antifungal testing procedure for *Fusarium* species against different antifungal drugs is described (Figure 1). 

### 9.1. Sample Preparation

Prior to AFST, and according to CLSI [23], *Fusarium* strains should be cultured on potato dextrose agar for 48 to 72 h at 35 °C and then until day seven at 25 °C for slowly growing species. However, Al-Hatmi et al. [59] indicated that incubation of *Fusarium* strains for 3–5 days at 27 °C is suitable for AFST.

### 9.2. Labeling of Materials

Working on a laboratory bench, preparing for this procedure by start labeling culture plates, *Fusarium* strains, preparing the dilution tubes, saline tween 20, Dimethyl sulfoxide (DMSO), (Sigma-Aldrich-Poole, Doorset, UK), Roswell Park Memorial Institute medium (RPMI) 1640, (Sigma-Aldrich-Poole), and a microtiter AST plate with the appropriate identifiers (e.g., patient name, medical record number). 

### 9.3. Laboratory Personnel Preparations

Appropriate personal protective equipment is necessary to work in the BSL-2. BSL-2 practices, containment, equipment and facilities are recommended, especially the wearing of a laboratory coat, safety glasses and disposable gloves; the gloves must be impervious to organic solvents.

### 9.4. Preparation of the Biological Safety Cabinet

Prepare the biological safety cabinet (BSC) by disinfecting the surface and placing a paper towel soaked with disinfectant approximately 6 inches from the air vent panel. Preferably, waste container should be placed on the left, and a small vortex on the right. Inoculating loops, cotton swabs, pipets and tips are placed beside the paper towel. A rack with the test fungi (on solid medium) should be placed on the paper towel.

### 9.5. Preparation of the Inoculum

Working in the BSL-2 cabinet, the cotton swabs are moistened with sterile saline with 0.005% Tween 20 and spores are harvested from the colonies on solid medium. Suspensions are made in saline with 0.005% Tween 20 (approximate 0.5 Mc Farland; equivalent to 1–5 × 10^6^ CFU/mL) standard. After allowing heavy particles to settle for three to five minutes, the upper homogeneous suspension is transferred to a sterile tube and vortexed for 15 s. The transmission is measured with a spectrophotometer at 530 nm that ranges from 0.15–0.17 (65%–70%T). The 1:50 (CLSI) inoculum dilution will be 2× (twofold) more concentrated than the density needed and 10× (EUCAST) approximately 0.4–5 × 10^4^ (CLSI) and 2–5 × 10^6^ (EUCAST).

### 9.6. Calibration of Spectrophotometer and Measuring Transmission

The transmission (T) option should be selected (“530 nm—100%T”) setting it as a blank using saline Tween 20. Once the blank is set then the cuvette with the sample suspension is placed in the cuvette holder with its smooth side to the front, and the lid is closed. The transmission of this particular suspension is measured “530 nm and measured %T”, it should be between 65−70 for *Fusarium* species.

### 9.7. Inoculation

The procedure involves preparing two-fold dilutions of the antifungal drugs. This can be achieved by dilution of antifungal in serial dilutions (e.g., 4, 8, 16, 32 and 64 μg/mL) in a liquid growth medium (RPMI 1640) dispensed in tubes containing a minimum volume of 2 mL (macrodilution) or with smaller volumes using 96-well micro-titration plates. Then, each well is inoculated with antifungals prepared in the same medium after dilution of standardized microbial suspension adjusted to 0.5 McFarland scale. Also the growth control wells are inoculated with 100 µL of sterile drug-free medium, and 100 µL of the same inoculum suspension. Following 48 h incubation at 35 °C, the microdilution plates are examined for visible *Fusarium* growth. The lowest concentration of antifungal that prevented growth represented the minimal inhibitory concentration (MIC). 

### 9.8. Interpretation of Results

Microdilution plates can be read manually using a mirror as an aid or with an automated plate reader such as the Vizion (TREK Diagnostic Systems, Cleveland, OH, USA). The plates can be examined as soon as 24 to 48 h after inoculation. The growth control wells are also examined to determine if they are positive (i.e., have a deposit of cells at the bottom of the well). If the growth control wells are not positive, the plate should re-incubated and re-examined (e.g., 24, 48 and 72 h) or until the growth control wells are positive.

The MIC of amphotericin B, voriconazole and the newer azoles is the well with the lowest concentration with 100% inhibition of growth (compared to the growth control well). In the case of echinocandins against *Fusarium* the minimal effective concentration (MEC) is determined. The MEC is defined as the lowest concentration of drug that leads to the abnormal growth of the fungus (small, rounded, and/or compact hyphal forms) in comparison to the control hyphal growth. ECVs determined by Espinel-Ingroff et al. [28] using CLSI methodology for *F. oxysporum* and *F. solani* species complexes and for *F. verticillioides* of the *fujikuroi* SC are as follows: for amphotericin B 4 μg/mL (*F. verticillioides*) and 8 μg/mL (*F. oxysporum* SC and *F. solani* SC); for posaconazole, 2 μg/mL (*F. verticillioides*), 8 μg/mL (*F. oxysporum* SC), and 32 μg/mL (*F. solani* SC); for voriconazole, 4 μg/mL (*F. verticillioides*), 16 μg/mL (*F. oxysporum* SC), and 32 μg/mL (*F. solani* SC); and for itraconazole, 32 μg/mL (*F. oxysporum* SC and *F. solani* SC). 

In general, there is a great variability in regard to their in vitro susceptibility to different antifungal agents. In 2003, a study from USA and Canada using a percentage of strains <1 µg/mL to indicate susceptibility showed 82% were susceptible to amphotericin B and 18% to voriconazole and posaconazole and no strains were considered susceptible to itraconazole and caspofungin [60]. In 2005, 57 strains of *Fusarium* from Mexico had MIC_90%_ >1 μg/mL for itraconazole, posaconazole and voriconazole [61]. In a study in Spain with 44 isolates of *Fusarium* spp. showed that *F. oxysporum*, *F. solani* and *F. verticillioides* were resistant to itraconazole, voriconazole and posaconazole with MIC_90%_ ≥8 µg/mL. Both *F. solani* and *F. verticillioides* had high MIC_90%_ to amphotericin ≥4 μg/mL whereas the MIC_90%_ for *F. oxysporum* was 1 μg/mL indicating susceptibility to amphotericin B [62]. One year later, another Spanish study, reported very high MICs for *F. oxysporum*, *F. proliferatum*, *F. solani* and *F. verticillioides* [10]. A study in the Netherlands in 2015 focused on *fujikuroi* complex and used molecular methods to identify species and then compare the identification to their antifungal patterns [11]. The species were as follows: 10 strains of *F. verticillioides*, 9 each of *F. proliferatum* and *F. sacchari*, 7 each of *F. acutatum*, *F. fujikuroi*, *F. napiforme* and *F. nygamai*, 6 each of *F. ananatum* and *F. thapsinum*, 5 of *F. anthophilum*, 4 of *F. andiyazi*, 3 of *F. subglutinans* and 1 of *F. temperatum.* Amphoterecin B was the most active drug with MICs ranging between 0.125 and 8 µg/mL. For fluconazole, itraconazole, and micafungin all strains showed high MIC/MEC values of ≥64, >16 and 8 µg/mL, respectively. The *Fusarium* strains tested in this study had variable susceptibilities to Amphoterecin B, voriconazole, posaconazole and isavuconazole, with MICs ranging between 0.125 and 8 µg/mL for amphotericin B, 0.5 to 8 µg/mL for voriconazole (one strain tested had a MIC of 16 µg/mL); 0.25 to ≥16 µg/mL for posaconazole and 1 to ≥16 µg/mL for isavuconazole [11]. The authors concluded that amphotericin B seemed to be the most active agent followed by voriconazole; however, they suggested that proper identification of species within the complex should be done in order to provide correct information for treatment especially *F. thapsinum* and *F. nygamai* because both species showed high MICs to the most drugs tested. Another study from India looked at 60 *Fusarium* isolates mainly from *F. solani* complex from keratitis cases and based on the observed MIC data in this study, authors reported that amphoterecin B and voriconazole had ≤4 µg/mL and natamycin ≤4 µg/mL are potential antifungal agents for the treatment of human keratomycosis caused by *Fusarium* [11]. Overall, *Fusarium* showed high MICs against fluconazole, itraconazole and echinocandins.

Since most strains have high MICs (intrinsic resistance) and since there is no breakpoints for susceptibility in the CLSI and EUCAST, we cannot determine susceptible/resistant, but different degrees of resistance at most can be determined in different strains. As the susceptibility profile is isolate dependent, antifungal susceptibility testing should be performed especially for any *Fusarium* involved in an invasive infection. Although susceptibility testing for *Fusarium* has lagged behind that for others like *Aspergillus* and *Candida*, much progress has been made over the past decade. Such knowledge should improve the clinician’s ability to select the best choice among the antifungal agents available. Further work needs to be done to correlate in vitro findings with in vivo, either using animal models or clinical outcomes. The collection of such data will allow for the establishment of interpretive breakpoints, which have already been established for *Aspergillus* and *Candida* species with some antifungal agents.

## 10. Conclusions

Prior to antifungal susceptibility testing, proper identification of the *Fusarium* strain is absolutely compulsory. Susceptibility testing should be performed in particular infection categories: invasive forms, infection in immunosuppressed individuals, upon therapeutic breakthrough infections, cases of keratitis or if antifungal treatment clinically failed. Susceptibility testing should be performed only if each analytical run includes the appropriate quality controls in accordance with the reference standards.
